# A Comprehensive Study on Light Signals of Opportunity for Subdecimetre Unmodulated Visible Light Positioning

**DOI:** 10.3390/s20195596

**Published:** 2020-09-29

**Authors:** Sander Bastiaens, Kenneth Deprez, Luc Martens, Wout Joseph, David Plets

**Affiliations:** WAVES, Department of Information Technology, Ghent University/imec, Technologiepark-Zwijnaarde 126, B-9052 Ghent, Belgium; Kenneth.Deprez@ugent.be (K.D.); Luc1.Martens@ugent.be (L.M.); Wout.Joseph@ugent.be (W.J.); David.Plets@ugent.be (D.P.)

**Keywords:** unmodulated visible light positioning, uVLP, visible light positioning, VLP, LED, received signal strength, localisation

## Abstract

Currently, visible light positioning (VLP) enabling an illumination infrastructure requires a costly retrofit. Intensity modulation systems not only necessitate changes to the internal LED driving module, but decrease the LEDs’ radiant flux as well. This hinders the infrastructure’s ability to meet the maintained illuminance standards. Ideally, the LEDs could be left unmodulated, i.e., unmodulated VLP (uVLP). uVLP systems, inherently low-cost, exploit the characteristics of the light signals of opportunity (LSOOP) to infer a position. In this paper, it is shown that proper signal processing allows using the LED’s characteristic frequency (CF) as a discriminative feature in photodiode (PD)-based received signal strength (RSS) uVLP. This manuscript investigates and compares the aptitude of (future) RSS-based uVLP and VLP systems in terms of their feasibility, cost and accuracy. It demonstrates that CF-based uVLP exhibits an acceptable loss of accuracy compared to (regular) VLP. For point source-like LEDs, uVLP only worsens the trilateration-based median $p_{50}$ and 90th percentile root-mean-square error $p_{90}$ from 5.3cm to 7.9cm (+50%) and from 9.6cm to 15.6cm (+62%), in the 4m × 4m room under consideration. A large experimental validation shows that employing a robust model-based fingerprinting localisation procedure, instead of trilateration, further boosts uVLP’s $p_{50}$ and $p_{90}$ accuracy to 5.0cm and 10.6cm. When collating with VLP’s $p_{50}=3.5\;cm$ and $p_{90}=6.8\;cm$, uVLP exhibits a comparable positioning performance at a significantly lower cost and at a higher maintained illuminance, all of which underline uVLP’s high adoption potential. With this work, a significant step is taken towards the development of an accurate and low-cost tracking system.

## 1. Introduction

Visible Light Positioning (VLP) is the latest indoor localisation technology, competing with, amongst others, ultra-wideband (UWB), Bluetooth Low Energy (BLE) and WiFi for a share of the booming Indoor Positioning and Indoor Navigation market. As a neophyte technology, VLP mainly situates itself in the research stage [1]. However, early (commercial) roll-outs have already appeared as well [2].

Innovation in VLP-based systems is driven by the promise of pairing a low cost to a high accuracy. The latter results from the sub-decimetre order positioning error reported in theoretical studies [3,4,5]. The practical evaluations of Li et al. [6] in a 2.5m by 2.84m by 2.5m area equipped with 7 Light-Emitting Diode (LED) lamps exhibited a 90th percentile root-mean-square error (rMSE) $p_{90}$ of approximately 25cm and 60cm, respectively, with and without the application of particle filter-based sensor fusion. A 1.9cm and a 16.1cm median positioning error was found in, respectively, a 3.3m by 2.1m laboratory and a 7.5m by 8m open foyer environment, when employing spring-relaxation-based positioning [7].

The inherent low cost of VLP systems should arise from them adding communication (and thus positioning) capability to the existing LED lighting infrastructure. Enabling this dual functionality is (1) the LEDs’ ability to imperceptibly switch between intensity levels at a high frequency i.e., intensity modulation (IM) [8,9] and (2) the ability to modulate the LED light’s polarisation [10,11]. In reality, VLP-enabling the (existing) illumination infrastructure requires a retrofitting step. In IM systems, hardware changes to the internal LED driving module are needed. In addition, modulating the driving current of the illumination LEDs decreases their radiant flux. In the frequency division multiplexing access (FDMA) scheme of De Lausnay et al. [12] the radiant flux $P_{t,i}$ effectively halves. The associated VLP system hence requires double the amount of LEDs for the same maintained illuminance, significantly augmenting the capital expenditure (capex). Polarisation-based VLP also reduces $P_{t,i}$ by at least 50% by employing linearly polarised light. Maturing advances in polarised LEDs [13] may render polarisation-based VLP more promising. Yang et al. obtain a decimetre 90th percentile positioning error $p_{90}$ with a 120 by 160 pixels Galaxy SII camera when operating in a 1.8m by 2.4m zone with 8 5V LED lamps covered with a twisted nematic liquid crystal polarisation modulator [11].

Employing the characteristics of light signals of opportunity (LSOOP), i.e., unmodulated VLP (uVLP), might pose a solution. In uVLP, the LED lamps remain unmodified, no modulation is performed. In [14], the authors treated 2 LSOOP localisation principles: (1) Georeferencing the measured (with a spectrometer) wavelength spectrum with respect to a database using correlation and (2) a LED lamp proximity-matching of the peaks of the total measured illuminance over distance relation. In [15], Amsters et al. input received signal strength (RSS) and encoder values in an iterated extended Kalman filter to navigate a photodiode (PD)-equipped mobile robot inside 10m by 10m room fitted out with a square 4 LED configuration. A simulation experiment showed that a single PD (and thus a single light measurement) not necessarily results in an unambiguous position, and that even with multiple PDs the positioning error keeps exceeding 50cm. In [16], the authors propose IDyLL, which combines dead-reckoning and light measurements to achieve mean location errors up to and exceeding 50cm. In [17], a tilted receiver on a rotator is able to 3D localise around a single LED. The previous works [14,15,16,17] all considered the total illuminance measured, i.e., the sum of all LEDs’ individual illuminance contributions. Their applicability depends on the presence of an illuminance gradient, which is minimised in practice as a uniform illuminance distribution is strived for.

Zhang et al in [18] exploit the inherent characteristic frequency (CF) (to demultiplex the individual contributions) of fluorescent lights with a commercial off-the-shelf smartphone to ensure a 37cm $p_{90}$ accurate positioning in 23.7m by 6.4m by 3.1m area. The CF originates from a resonance in the fluorescent light’s inverter.

### 1.1. Problem Statement

Though fluorescent lights’ low cost and high availability restrain them from phasing out rapidly, the solid-state lighting (r)evolution will see the illumination market transition to LED technology. While mid/high-power LED deployments differ extensively from their fluorescent light counterparts, their constant current driver induces resonance frequencies as well [19]. In LED drivers, additional output capacitors tend to suppress the non-DC frequencies, which in turn attenuates the CFs significantly. To what extent the attenuated CFs can still be employed for accurate positioning has not yet been studied.

### 1.2. Paper Content

This paper studies whether the LED’s characteristic frequency can serve as a discriminative feature in PD-based received signal strength (RSS) uVLP. Usually, in RSS-based VLP, LEDs are intensity modulated for the transmission of signals that are demultiplexable at the receiver. In case of frequency division multiple access [12], each LED is purposely assigned a unique and distinguishable frequency. The premise of RSS-based uVLP is to use the LEDs’ characteristic frequency to separate each individual LED’s RSS contribution.

Hereto, first, the frequency (pseudo)spectrum of 5 different types of LED - LED driver topologies, as obtained via MUltiple SIgnal Classification (MUSIC) [20], is studied as to ascertain the (potential) presence of a unique CF. The (frequency) dependence and/or stability of the CF with respect to driving current amplitude or pulse-width modulation dimming is reported. Secondly, based on 2 large datasets collected, the positioning performance of uVLP and regular VLP is experimentally compared in a 3.95m by 3.95m by 2.25m (length × width × height) room equipped with a 4-LED constellations [21]. Finally, simulations are performed to evaluate the aptitude of uVLP in higher-ceiling (i.e., industrial) environments and to identify the minimal CF magnitude required for accurate positioning in noisy environments.

The main contributions of this paper are:

- A measurement-based study towards the manifestation of a characteristic frequency with 5 different LED light and LED driver combinations.
- An experimental positioning performance comparison between uVLP and (regular) VLP positioning, for different demodulation and positioning procedures.
- The introduction of more robust versions of the model-based fingerprinting approach of [22].
- Simulations, matching and extending the experimental results, to investigate the feasibility of uVLP in environments with higher ceilings.

Inherently low-cost, RSS-based uVLP has a significant potential for application in next-generation tracking systems. This manuscript’s aim is to demonstrate CF-based uVLP’s feasibility and to optimise its positioning accuracy in order to reach that potential.

Compared to the total illuminance-based approaches [14,15,16,17], which are unable to cope with the minimised illuminance gradients found in practice, CF-based uVLP attains more accurate localisation as a consequence of it employing the per LED RSS values. This work differentiates from [18] as it focusses on uVLP systems with LEDs and PDs (versus with fluorescent lights and camera). It is also the first work that studies CF-based uVLP positioning performance with detailed measurement datasets.

## 2. Materials and Methods

### 2.1. Characteristic Frequency

**Figure 1:**
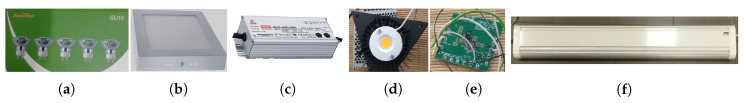
Illustration of the LED fixtures and drivers considered: (**a**) KLLUG-511, (**b**) 18WLEDQSM, (**c**) HLG-40H-48A, (**d**) BXRE-35E2000-C-73, (**e**) LTM8005 Demo Board and (**f**) E4010/LED1N060D.

LED lamps appear in conjunction with LED drivers of different topologies and complexity, depending on the envisioned cost and application [19]. As a result, the LED lamp roll-outs vary in radiated waveform distortion (and associated spectrum interharmonics), (grid) emission characteristics and impedance level [23,24]. The radiated waveform of LEDs is generally more miscellaneous than for their fluorescent counterparts [24]. Interestingly, within LEDs of the same type as well, a substantial variation in their emitted radiant spectrum is present, which allows the identification of a characteristic frequency (CF) for each LED. This CF can be exploited to demodulate the individual photovoltage contributions $V_{PD,i}\left(t\right),i=1\ldots N$ of each of the *N* LEDs from the total photovoltage $V_{PD}\left(t\right)$ generated at the PD-receiver, without having to modulate or even modify the LEDs. The next section measures $V_{PD}\left(t\right)$ for various LED lamp types and topologies (all of which are depicted in Figure 1) in order to study the manifestation and the magnitude of the CF.

### 2.2. Measuring the Characteristic Frequency

A Thorlabs PDA36A2 (https://www.thorlabs.com/thorproduct.cfm?partnumber=PDA36A2) is placed horizontally opposite to the LED under consideration at 50cm distance, unless otherwise specified. The PD in combination with National Instrument’s USB-6212 DAQ device measures $V_{PD}\left(t\right)$ (100 times for a number of samples $N_{a}=51.2\;kS$ at a rate $f_{S}=256\;k\mathrm{Hz}$ and with a $1.51\times 10^{3}\;V\;/\;A$ transimpedance gain) [21]. The LEDs’ CF is identified via peak detection on the 100 times averaged pseudospectrum, obtained by running the well-known algorithm MUltiple SIgnal Classification (MUSIC) [20] on the $V_{PD}\left(t\right)$ time series. MUSIC’s subspace order is set to 10. The five LED driver - LED combinations of Figure 1 are investigated.

#### 2.2.1. Single-Phase Bridge Rectifier-Based LED Topology

**Figure 2:**
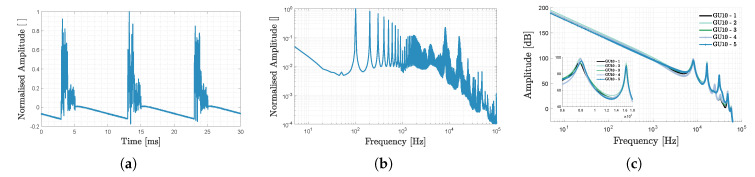
KLLUG-511 appertaining (**a**) mean-subtracted normalised $V_{PD,i}\left(t\right)$ time domain waveform, (**b**) first harmonic-normalised fast Fourier transform (FFT) spectrum and (**c**) MUSIC’s output pseudospectrum for 5 LEDs.

The first LED to be investigated is a GU10-connected 4W KLLUG-511 LED lamp of the Ascher make (see Figure 1a), meant to replace the 50W halogen bulbs. It outputs 400lm at 2900K and consists of individual LED surface mounted devices. The passive LED driving module lacks a constant current integrated circuit, and mainly operates by linking a single-phase bridge rectifier to the LED’s terminals. The mean-subtracted normalised $V_{PD}\left(t\right)$ time domain waveform, the first harmonic-normalised fast Fourier transform (FFT) spectrum and MUSIC’s output pseudospectrum (of five LEDs from the same batch) are depicted in Figure 2.

Figure 2a boasts a non-smooth and oscillation-filled waveform, while (b) shows the abundance of harmonics of the 100Hz double mains frequency [24] clearly visible in the below 2kHz range [25]. Figure 2c shows the presence of higher-frequency resonances (e.g., around 8 or 16kHz), which could feature as CF. The pseudo-spectrum peaks are rounded and the small inter-LED spread (<20Hz), renders it difficult to define a unique and robust CF.

#### 2.2.2. 18wledqsm LED Panel

**Figure 3:**
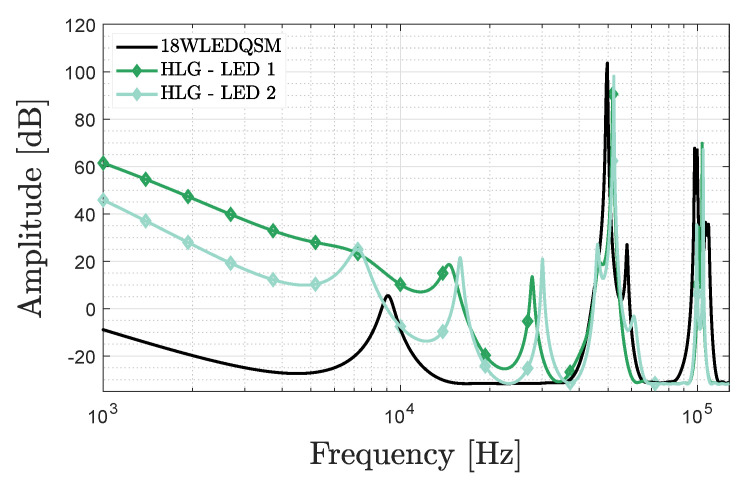
MUSIC pseudospectrum versus frequency of the 18WLEDQSM LED panel (black curve) and for a single HLG-40H-48A module driving two different BXRE chip on board (COB) LEDs (in green).

The pseudospectrum of the floodlight LED panel 18WLEDQSM (depicted in Figure 1b) with a Dark Energy LED driver is shown in black in Figure 3. The LED driver module has a buck constant current driver and an isolation transformer as main additional building blocks. It can be remarked that the distortions are found higher-up in frequency, and that a CF is present around 49.75kHz. The current waveform is smoother than for the previous LED.

#### 2.2.3. BXRE-35E COB LEDs

Chip on board (COB) LEDs, of which BXRE-35E2000-C-73 (https://www.bridgelux.com/sites/default/files/resource_media/Bridgelux%20DS101%20Gen%207%20V13%20Array%20Data%20Sheet%2020190930%20Rev%20N.pdf) is the considered example shown in Figure 1d, are favoured in VLP as their resemblance to Lambertian radiating point sources allows an easy localisation [21]. Coupling 2 different BXRE LEDs with the same high-end HLG-40H-48A (https://www.meanwellweb.com/en-gb/ac-dc-single-output-led-driver-mix-mode-cv-cc-with-hlg--40h--48a) (Figure 1c) constant current regulator of MEAN WELL results in the green pseudospectra of Figure 3. Both LEDs show a distinct CF at, respectively, 51.84kHz and 52.29kHz.

**Figure 4:**
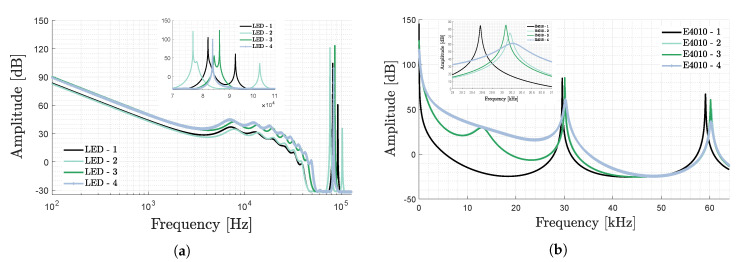
MUSIC pseudospectrum versus frequency on (**a**) a logarithmic scale of 4 LTM8005 demo boards each driving a BXRE COB LED (subspace order 20), and (**b**) on a linear scale of 4 ETAP E410 LED armatures.

As this manuscript’s goal is to compare positioning based on the CF, i.e., unmodulated VLP (uVLP) with dedicated VLP, an identical LED driver is to be used for both to ensure a fair comparison. Commercial VLP drivers are however not readily available. As such, in this paper, the LTM8005 Demo Board (https://www.analog.com/en/products/ltm8005.html#product-overview) (Figure 1e) is chosen to drive the BXRE-35E2000-C-73 COBs in either AC or DC. The DC pseudospectrum for the N=4 LEDs, measured on the ground in the positioning setup of Section 2.6, is given in Figure 4a. The found CFs amount to 76.57kHz, 81.82kHz, 83.49kHz and 86.09kHz.

#### 2.2.4. ETAP’S E4010 LED Armature

Figure 4b shows the pseudospectrum belonging to 4 industry-grade ETAP (https://www.etaplighting.com/en) narrow-angle E4010/LED1N060D (https://www.etaplighting.com/en/series/e4/e4010led1n060d) LED luminaires (shown in Figure 1f). Each LED armature has a distinct peak around 30kHz that can serve as CF. The minimal spacing between the LEDs’ CF is 80Hz.

In this Section 2.2, the scope was limited to LED lamps on account of LEDs being both the ‘illumination sources of the future’ [26] and the lights typically used in VLP. The latter, which can (partially) be attributed to the LEDs supporting higher modulation frequencies [2], allows comparing the positioning accuracy of PD-based uVLP and standard VLP.

### 2.3. Applicability of the Characteristic Frequency

The CF measurements of the 4 constant current LEDs permit drawing 4 conclusions. First, the CF is dependent on the individual LED lamp’s characteristics. Second, all constant current driven LED lamps exhibit a unique CF that is furthermore prominent enough to use as a basis for positioning, provided that the CF is robust. The robustness will be demonstrated in the next Section 2.3.1 and Section 2.3.2. The KLLUG-511 LED lamp, characterised by the absence of a constant current driver, is ill-suited for CF-based uVLP. Third, with the CFs up to and exceeding 80kHz, uVLP either dictates a significant rate $f_{S}$ or necessitates the use of the CF aliases. For the former, $f_{S}$ should exceed 2 times the maximum CF, provided the absence of higher frequency components that could alias into the CF range. The CF-range also imposes a minimal 3dB receiver bandwidth, which in turn puts restrictions on the receiver chain due to the inherent bandwidth - gain trade-off (of the involved (transimpedance) amplifier circuits). Depending on the photodiode’s junction capacitance, PD-bootstrapping or multiple gain stages may need to be used. Interestingly, the CF of a fluorescent lamp is also found to be larger than 80kHz. It is located in the 80–160 kHz range [18]. Hence, camera-based uVLP needs the receiver camera’s sampling process to be optimised in order to reach the required bandwidth and $f_{S}$ as well [18].

Finally, as inter-CF spreads are found to be as limited as 80Hz, CF-based uVLP will require a fine frequency resolution. As a consequence of the $f_{S}$ requirement of conclusion three, the number of recorded samples $N_{a}$ will be more substantial than generally is the case for VLP. Unfortunately, as a higher $N_{a}$ dictates a lower refresh rate, it is more difficult uVLP-based tracking system to be real-time.

#### 2.3.1. Stability of the CF over Time

**Figure 5:**
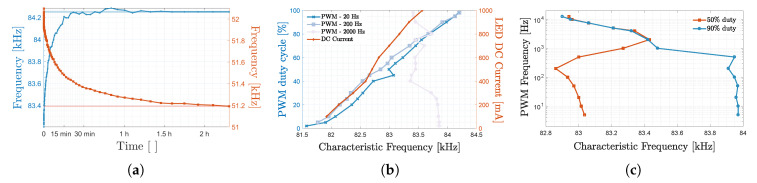
Characterising the characteristic frequency (CF) as a function of (**a**) time and compatibility with dimming with respect to (**b**) the average LED current and the (**c**) pulse-width modulation (PWM) frequency, for a BXRE chip on board (COB) LED measured on the ground in the positioning setup of Section 2.6.

For uVLP to be able to ensure accurate and consistent localisation, it requires a certain robustness i.e., frequency stability of the LEDs’ CF. Figure 5a portrays a time-lapse of the CF for the same BXRE LED, coupled to the HLG-40H-48A and LTM8005 LED driver. As was reported for fluorescent lamps by Zhang et al. in [18], the LEDs CF shows a significant start-up behaviour that lasts for more than an hour. At the end of this start-up, the LED current magnitude and temperature stabilise. Importantly though, in stable regime, the standard deviation on the CF equals a workable 6.47Hz and 8.47Hz, respectively. Discontinuing and reapplying the LED current, i.e., a new start-up, yields a comparable (within a standard deviation tolerance) CF as before. Hence, the CF remains stable across switching the LEDs ‘off’ and ‘on’. It should be noted that temporal variation is not only present in the CF, but also in its spectral magnitude. Possible temperature-dependencies of the nominal value and temporal variation of the CF requires further study.

#### 2.3.2. Stability of the CF with Light Dimming

As stated in the introduction, VLP intends to leverage the existing illumination infrastructure. Important herewith is to remain compatible with the in-place interfaces (e.g., Digital Addressable Lighting Interface (DALI)) and to support LED driving current dimming via amplitude and/or pulse-width modulation. Ideally, a LED’s CF would be unaffected by variations in its LED current.

However, Figure 5b,c show that this is not the case. In (b), it displays (in red) an approximately linear relation between the CF and the driving current. For pulse-width modulation (PWM) frequencies up to 200Hz, increasing the duty cycle, and thus the average LED driving current, also rises the CF. PWM frequencies exceeding the 2kHz threshold exhibit a more peaky pseudospectrum, requiring a higher subspace order distinguish a CF. Identifying the CF as the frequency peak closest to the DC CF value, shows the higher-up PWM frequencies remaining more constant with increasing duty cycle (see Figure 5c).

A frequency shifting CF does not necessarily mean that uVLP is incompatible with PWM dimming i.e., as long the LEDs’ $V_{PD}\left(t\right)$ contributions are demultiplexed to the correct LED. In RSS-based uVLP, the receiver needs to be aware of the varying duty cycle as the CF’s magnitude and frequency can change. This change can be coped with by either modelling the shifts or via (re)calibration.

### 2.4. Demodulation

**Figure 6:**
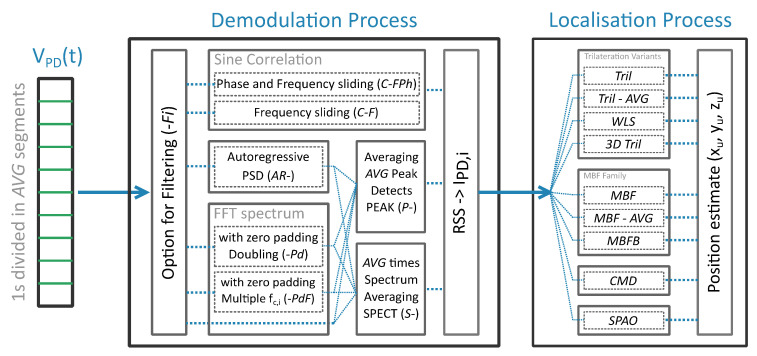
Flowchart representation of the (unmodulated) visible light positioning (u)VLP demodulation and positioning chain. The acronyms of Section 2.4 and Section 2.5 are also gathered in a table in Abbreviations.

The positioning methods of the next Section 2.5 employ the set of per LED received radiant powers $\{P_{R,i}\},\;i=1..N$ as input, i.e., as RSS values. The individual power contributions $\left\{P_{R,i}\right\}$ are derived from their photovoltage variants $\left\{V_{PD,i}\right\}$ after division by the transimpedance gain (leading to the photocurrent magnitudes $\left\{I_{PD,i}\right\}$) and subsequently by the receiver responsivity [21]. $\left\{V_{PD,i}\right\}$ are in turn demodulated from the total photocurrent $V_{PD}\left(t\right)$. Figure 6 visualises the receiver demodulation and positioning methods to be described below, the names of which are also collected in Abbreviations in list format.

Three demodulation methods are considered: (i) the FFT spectrum-based magnitude method from [12], (ii) sliding frequency window correlation, and (iii) the identification method of (i) applied on the (autoregressive) Yule-Walker method-based power spectral density (PSD) spectrum. In all three methods, the LED contributions are sought at and around the frequencies $\left\{f_{c,i}\right\}$, which either equals the (first harmonic) modulation frequency (in case of VLP) or the characteristic frequency (in case of uVLP). Due to temporal variation in the CF (see Section 2.1), methods (i) and (iii) first perform a peak detect in the neighbourhood 2Δf of the nominal $f_{c,i}$ i.e., $\left[f_{c,i}-\Delta f,\;f_{c,i}+\Delta f\right]$. In (ii), the maximum of all correlations of $V_{PD}\left(t\right)$ and the sinusoids with $f_{c,i}\in \left[f_{c,i}-\Delta f,\;f_{c,i}+\Delta f\right]$ is computed. (ii) is denoted by C-F. C-FPh is an extended version of C-F with an additional slide over the phase angle θ. For uVLP and VLP, Δf=100Hz (in steps of 10Hz) and Δf=15Hz (in steps of 2Hz), respectively.

Prior to demodulation, the $V_{PD,i}\left(t\right)$ time signal, spanning 1s, is subdivided in AVG segments to average $\left\{V_{PD,i}\right\}$. Two averaging methods are discerned: (1) the complex spectrum is averaged prior to peak detection of $\left\{V_{PD,i}\right\}$ on the amplitude spectrum, and (2) the peak detected $\left\{V_{PD,i}\right\}$ of each of the segments is averaged. For (i), methods (1) and (2) are, respectively, named SPECT (with abbreviation *S*) and PEAK (with abbreviation *P*). During (iii), (1) and (2) are designated by AR-S and AR-P.

The last two demodulation techniques considered entail FFT-based zero padding (a) as to double the photovoltage signal’s length (denoted with suffix Pd) and (b) as to obtain a per LED FFT period that is a multiple of $f_{c,i}$ (denoted with suffix PdF). Striving for coherent sampling, PdF consists of 3 steps for each LED: a peak detect on the ‘standard’ FFT amplitude spectrum to determine the instantaneous CF, a recompute of the FFT-spectrum based on the new zero padded signal, and a second peak detect. PdF represents a limited-complexity version of finding the best match in terms of the CF and the amount of zero-padded samples.

### 2.5. Positioning Procedure

Ensuring an accurate localisation of the unknown receiver position denoted by $\left(x_{u},y_{u},z_{u}\right)$, both during uVLP and VLP, requires an off-line site survey to accurately chart the LEDs’ coordinates $\left(x_{S,i},\;y_{S,i},\;z_{S,i}\right)$, radiant powers $P_{t,i}$ and $f_{c,i}$, i=1..N. $\left(x_{u},y_{u},z_{u}\right)$ is estimated by means of the localisation algorithms detailed in the subsequent parts, all of which require a channel model as input. In this work, as is frequent in literature [2], the infrared propagation models of Kahn et al. [27] will serve as the VLP channel models for converting the $\left\{P_{R,i}\right\}$ sets into positioning estimates. The description of Kahn’s models is the same as in our previous work [21]. A SQ receiver angular acceptance is assumed [21]. For the various positioning algorithms to follow, the *K* parameter (with K≤N) selects, in terms of a descending $P_{R,i}/P_{t,i}$ order, which (sub)set of $\{P_{R,i}\},\;i=1..N$ is used to determine the position estimate.

#### 2.5.1. Trilateration-Based Localisation

This work will compare the positioning performance of 4 trilateration-based 2D RSS algorithms. (1) The baseline trilateration algorithm, denoted by Tril, computes a location estimate of $\left(x_{u},y_{u},z_{u}\right)$ by (least-squares) solving the linear system relating $\left(x_{S,i},\;y_{S,i},\;z_{S,i}\right)$ to the LED-receiver distance estimates $d_{i}$ [28]. The system only accounts for the K=3 LEDs with smallest $d_{i}$. (2) To increase robustness, Tril - AVG averages the location estimate obtained via Tril for the 4 LED combinations of 3 LEDs. (3) WLS employs a singular value decomposition to weigh the LEDs’ trilateration contributions as outlined in [29]. (4) 2D localisation 3D Tril by discarding the height estimation from the 3D trilateration approach detailed in [30].

$d_{i}$ is directly obtained from $P_{R,i}$ by inverting the VLP channel model. Requiring an invertible channel model has as drawbacks that it restricts both the LED radiation pattern and receiver acceptance model, and that it hinders the modelling of non-line-of-sight propagation. Both lead to a significantly degraded positioning performance [21,28]. In trilateration, the LEDs’ radiation pattern is approximated to be Lambertian. The best fitting Lambertian order of the Lambertian-like BXRE-35E2000-C-73 equals 1.14 [21].

#### 2.5.2. Cayley-Menger Determinant Localisation

Positioning based on a geometrical formulation, as opposed to the above algebraic formulation, leads to the Cayley-Menger Determinant-based localisation procedure (5) CMD of [31]. This paper studies CMD in 2D.

#### 2.5.3. Model-Based Fingerprinting Localisation

Model-based fingerprinting (MBF (6)) RSS VLP initially computes a propagation map (according to the VLP channel model), holding the expected RSS values per LED, i.e., $\left\{P_{R,i}\right\}$, for all locations on a predefined positioning grid. The propagation map accounts for the LEDs’ C0/C90 photometric diagram [21]. Upon a $\left\{P_{R,i}\right\}$ measurement, the grid position that has the minimal difference, in terms of a cost function, between the modelled $\left\{P_{R,i}\right\}$ and measured $\left\{\hat{P_{R,i}}\right\}$ is taken to be the positioning estimate. The following cost function C(x,y) is utilised (chosen based on later experimental results):

(1) $$C\left(x,y\right)=\sum_{i=1}^{K}{\left(1-\frac{P_{R,i}}{\hat{P_{R,i}}}\right)}^{2}\;.$$

(7) MBF-AVG is analogously defined as (2). This paper also proposes an MBF variant, namely (8) MBFB, to better cope with measurement variance of uVLP. In MBFB, the location estimate is taken to be the mean of all grid coordinates for which the C(x,y) is smaller than the *p*th percentile of C(x,y), rather than C(x,y)’s minimum was in MBF. MBF and MBFB are similar to the (K-) nearest neighbours (KNN) algorithm. However, a distinction is made between both on the grounds that in MBF and MBFB the fingerprinting database is being generated by computing a predefined channel model, while for KNN it is composed of measurements. An advantage of the MBF-approach is that upon positioning environment changeover, the fingerprints can be scaled or recomputed via the propagation model.

#### 2.5.4. Simultaneous Positioning and Orientating

This paper also reports the 2D positioning performance achieved with the Simultaneous Positioning and Orientating SPAO (9) approach of Zhou et al. [32]. 10 iterations are performed, and the (bounded) receiver orientation and z-coordinate are finally discarded. 3D Tril’s output is taken as the initial position.

### 2.6. Positioning Setup

**Figure 7:**
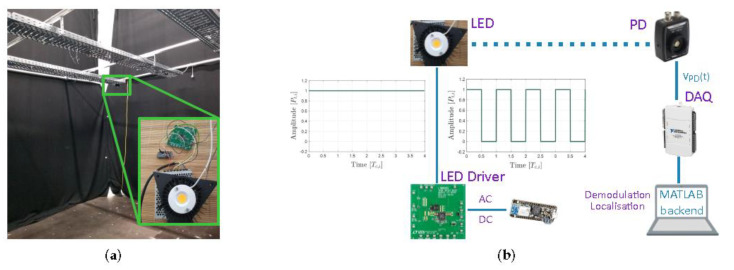
Illustration of the COB (u)VLP (visible light positioning) (**a**) lab setup and (**b**) schematic system overview.

The associated positioning performance is verified in our VLP lab [21], depicted in Figure 7a. Surrounded by black cloths to minimise reflections, the PDA36A2-based receiver (see Section 2.1) traverses a 2D plane that is situated 2.25m below the LED plane ($\left\{z_{S,i}\right\}=\left[2.242,\;2.252,\;2.247,\;2.250\right]\;\left(m\right)$). $I_{PD,i}\;\left(t\right)$, which is derived from $V_{PD,i}\;\left(t\right)$ by division with the (new) transimpedance gain $1.51\;\times \;10^{5}\;V\;/\;A$ is measured (during 1s at a rate $f_{S}=256\;k\mathrm{Hz}$) every 2.5cm across the ground plane and sequentially $1\;m^{2}$ at the time via Velmex’ BiSlides [21]. The photocurrent RSS values $I_{PD,i}$ are then subsequently obtained from the $I_{PD,i}\;\left(t\right)$ time waveforms as outlined in Section 2.4. The LED plane is occupied by N=4 LTM8005-connected BXRE-35E2000-C-73 LEDs placed in a rectangle: $\left\{x_{S,i}\right\}=\left[-1.13,\;-1.12,\;1.17,\;1.15\right]\;\left(m\right)$ and $\left\{y_{S,i}\right\}=\left[-1.41,\;1.44,\;-1.43,\;1.4\right]\;\left(m\right)$. Both LED types are driven to transmit either DC light or 50% duty cycle pulse train intensity modulated light. In AC regime, the LTM8005 driver’s frequency $f_{c,i}$ is dictated over WiFi via the Adafruit Feather M0 WIFI w/ATWINC1500 (https://www.adafruit.com/product/3010) module to satisfy: $f_{c,i}=2^{i-1}f_{0}$ [12] with $f_{0}=500\;\mathrm{Hz}$ exceeding the flicker threshold. The root-Mean-Square Error (rMSE) is the metric used to evaluate the positioning. Figure 7b provides a schematic overview of the employed VLP system.

#### $P_{T,I}$ and $F_{C,I}$ Calibration

$\left\{f_{c,i}\right\}$ are calibrated directly underneath each LED, with a single measurement. The nominal $P_{t,i}$ is computed by taking the mean of $P_{t,i}\;\cdot \;{\left(MMd_{i}\;/\;z_{S,i}\right)}^{3}$ for all grid points within a 20cm radius of ${\mathrm{LED}}_{i}$’s projection. For uVLP and VLP, the (rounded) mean $M\;\cdot \;P_{t,i}\;\cdot \;R_{P}\left(0\right)$, respectively, amounts to 0.04A and 5A (to be used in Section 4). The actual $M\;\cdot \;P_{t,i}\;\cdot \;R_{P}\left(0\right)$ values however (slightly) depend on the employed demodulation strategy (Section 2.4).

## 3. Experimental Results

This experimental section is devoted to studying the feasibility of CF-based uVLP, and to comparing the positioning performance of uVLP and VLP. As VLP localisation generally entails performing FFT-based demodulation [12], the latter is the demodulation technique discussed in the first Section 3.1 and Section 3.2. Later, Section 3.3 demonstrates the influence of the employed demodulation on both (u)VLP and whether it can boost the positioning accuracy.

### 3.1. $I_{Pd,I}$ Propagation

**Figure 8:**
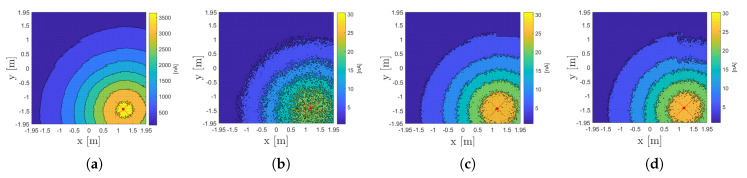
Distribution of $I_{PD,3}$, which is measured in $157^{2}$ grid points, for (**a**) VLP with AVG=1 and SPECT fast Fourier transform (FFT)-based demodulation and for uVLP with AVG =10 with, respectively, (**b**) SPECT-based, (**c**) PEAK-based and (**d**) correlation (C-FPh)-based demodulation.

Figure 8 shows a contour plot of the SPECT demodulated photocurrent contributions $I_{PD,i}$ of LED 3 (i.e., $I_{PD,3}$) for (a) VLP and (b) uVLP. The difference in both the absolute $I_{PD,i}$ magnitude and the (erratic) regularity of the $I_{PD,i}$ distribution between (u)VLP and VLP is clearly visible. It should be noted that during FFT-demodulation, the uVLP’s $I_{PD,i}$ is similarly scaled to the VLP’s $I_{PD,i}$, i.e., with π instead of 4, to show the relative RSS difference between VLP and uVLP at the receiver. π and 4 are the scaling factors of the first Fourier coefficient for a square and sine wave, respectively [12].

Now considering Figure 8b,c, appertaining to SPECT and PEAK, allows to conclude that uVLP’s PEAK $I_{PD,i}$ distribution exhibits less noisy, i.e., capricious behaviour than SPECT’s. In other words, PEAK exhibits a larger signal-to-noise-ratio (SNR) than SPECT. An explanation is found in the temporal instability of the (instantaneous) CF (see Section 2.3.1). As a consequence of a stable $f_{c,i}$, VLP PEAK and VLP SPECT yield the exact same $I_{PD,i}$. As SPECT is of lower complexity, it is therefore the designated FFT demodulation technique (for VLP). Finally, comparing Figure 8a with Figure 8b/Figure 8c permits concluding that uVLP’s $I_{PD,i}$ is 2 orders of magnitude smaller than VLP’s. Figure 8d also depicts the uVLP correlation-based $I_{PD,i}$ as showing a comparable regularity to PEAK. Correlation-based uVLP is treated more in detail later on.

### 3.2. Fft-Based Localisation

The subsequent parts of this section study FFT-based demodulation and characterise the influence of AVG.

#### 3.2.1. Tuning the Positioning Algorithm Parameters

First, the positioning algorithms of Section 2.5 need to be fully specified. MBFB, whose performance depends on the percentile *p*, is started with. As a consequence of the convexity of the MBFB $p-p_{75}$ relation (with $p_{75}$ being the 75th percentile rMSE) associated with each demodulation configuration (of Figure 8), the optimal *p* can be determined to equal *p* = 0.02%. After the $p-p_{75}$ infliction point (for *p* values exceeding 1%), an increasing *p* introduces errors at the room’s corners where the centre of gravity pulls MBFB’s location estimates inwards. Lowering the SNR (e.g., to an SNR comparable or lower than uVLP SPECT’s), shift the optimal *p* to a larger magnitude. p=0.02 corresponds to model-based KNN with 5 neighbours, which has been checked to outperform both KNN with 4 and 6 neighbours.

The parameter *K* denotes whether per grid point the 3 LEDs with the largest $P_{R,i}\;/\;P_{t,i}$ are selected, or all 4 LEDs are employed, for positioning. For MBFB, it turns out that K=3 works superiorly to K=4 for the demodulation configurations showing a higher SNR than uVLP SPECT. In fact, K=3 is also optimal (and is hence applied) for all positioning algorithms that do not perform a 3D localisation (except WLS). In 3D Tril, the height search range is limited to 2.2–2.7 m to avoid position ambiguities [33].

#### 3.2.2. AVG’s Frequency Resolution and Averaging Trade-Off

Now that the positioning algorithms are tuned, this part discusses the trade-off between the frequency domain resolution and the averaging amount during FFT-based (u)VLP. As stated in Section 2.4, per grid location, the 1s measurement duration is subdivided into AVG time domain segments (of length $N_{a}=N_{s}\;/\;AVG$ with $N_{s}=256\;kS$ the total sample count). AVG also denotes the FFT bin separation i.e., the frequency resolution. Hence, an upper bound AVG is instilled by the minimum $f_{c,i}$ separation of the LEDs.

**Figure 9:**
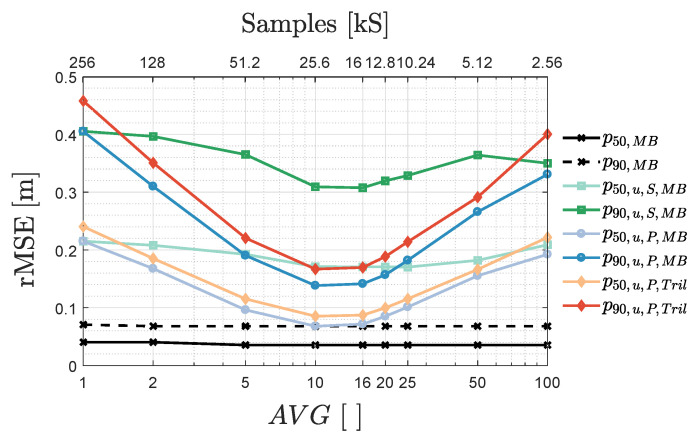
Influence of the AVG parameter on the $p_{50}$ and $p_{90}$ found both during FFT demodulation- and MBFB-based positioning in VLP (in black), uVLP SPECT (subscript ‘u,S’, in green) and PEAK systems (‘u,P’, in blue), and during trilateration (Tril)-based uVLP PEAK (in the red shades). ‘MBFB’ is abbreviated to ‘MB’.

Figure 9 shows a different AVG dependence of the 50th percentile $p_{50}$ and 90th percentile $p_{90}$ rMSE for VLP, uVLP PEAK and SPECT. In VLP, once AVG≥5, the localisation performance remains constant due to its large SNR. Its $p_{50}$/$p_{90}$ are subsequently dominated by consistent errors (treated later on in Section 3.3.3).

Figure 9 also illustrates the significant discrepancy between uVLP PEAK and SPECT. Better in accounting for instantaneous CF (magnitude)-instability, PEAK’s $AVG-p_{50}$/$p_{90}$ curves are convex and exhibit distinct minima. For AVG larger than the optimal AVG, issues in resolving the correct $f_{c,i}$ (magnitude) augment the $p_{50}$ and $p_{90}$. For uVLP SPECT, which is (vastly) outperformed by PEAK for AVG>1 and AVG<100, the $AVG-p_{50}$/$p_{90}$ curves vary more gradually. The $p_{50}$/$p_{90}$ curves appertaining to uVLP SPECT show a minimum at AVG=16, while AVG=10 minimises PEAK’s $p_{50}$ and $p_{90}$. It should be noted that uVLP SPECT’s $p_{50}$/$p_{90}$ can be lowered by fine-tuning MBFB’s *p* parameter (see Section 3.2.1). From Figure 9, it is also clear that the cost-effectiveness of PEAK uVLP does come at a $p_{50}$/$p_{90}$ cost when comparing with VLP systems.

Finally, MBFB outperforms Tril by 15.8%/16.8% and 11.2%/11.3% in terms of their $p_{50}$/$p_{90}$, respectively, for uVLP PEAK and SPECT. Tril exhibits the same AVG behaviour as MBFB, supporting this section’s conclusions.

### 3.3. Demodulation Technique and (U)VLP Accuracy

The above analysis demonstrated the feasibility of uVLP, potentially supplemented with location tracking/filtering techniques [34], for indoor localisation applications that require positioning accuracies in the 10–30 cm range. Several applications, such as the indoor navigation of robots require positioning errors in the subdecimetre range.

This Section 3.3 looks at the other demodulation methods of Section 2.4 (with or without additional filtering techniques) in an attempt to further boost (u)VLP’s positioning rMSE. This analysis is performed for AVG=10 and $N_{a}=25.6\;kS$ as to discern between 10Hz-spread $f_{c,i}$.

#### 3.3.1. Influence of Demodulation on (U)VLP Rmse

**Figure 10:**
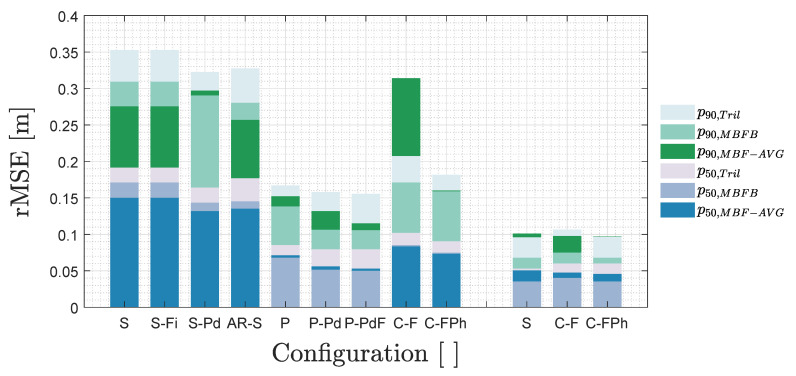
A bar chart representing the $p_{50}$ and $p_{90}$ for Tril, MBF-AVG and MBFB for various (u)VLP demodulation configurations. The left 9 bars represent uVLP configurations, while the right 3 bars constitute VLP-based demodulation schemes. The etymology of the configuration names is clarified in Figure 6.

Figure 10 shows a bar chart representation of the $p_{50}$ and $p_{90}$ for 3 positioning algorithms (namely Tril, MBF-AVG and MBFB) for (the first) 9 uVLP and (the latter) 3 VLP demodulation configurations.

The first configuration SPECT (*S*) serves as a uVLP rMSE baseline. Its associated $p_{50}$/$p_{90}$ in order amounts to 15.0cm/27.5cm for MBF-AVG, to 17.1cm/30.9cm for MBFB and to 19.1cm/35.3cm for Tril. Configuration 2, S-Fi demonstrates the limited added benefit of using time/frequency-based filtering. Filtering based on the maximal overlap discrete wavelet transform with the Daubechies 4 wavelet improves the SPECT-based MBF-AVG scores but with a few millimetres. Utilising other filters, such as the savitzky-golay filter, in combination with either demodulation technique, also did not effectuate much rMSE gain. Zero padding before SPECT demodulation, namely S-Pd, ameliorates the $p_{50}$ of MBF-AVG, MBFB and Tril, respectively, by 1.8cm, 2.8cm and 2.8cm. For MBF-AVG, this comes at the cost of a (slight) $p_{90}$ increase. In configuration 4 with the autoregression-based demodulation (AR-S), MBF-AVG still scores best and even better than for SPECT. It rates at 13.5cm/25.7cm. AR-S’s PSD estimations seemingly cope better with the CF variance.

As stated in Section 3.1 and Section 3.2.1, utilising PEAK FFT-demodulation drastically reduces the positioning rMSE to 6.8cm/13.8cm for MBFB, to 7.1cm/15.2cm for MBF-AVG and to 8.5cm/16.7cm for Tril. The rMSE values represent relative improvements over the SPECT baseline’s of 60.2%/55.3%, 52.5%/44.7% and 55.4%/52.6%, respectively. P is the first demodulation technique that delivers its best positioning estimates in conjunction with MBFB instead of MBF-AVG. In fact, all techniques that deliver positioning estimates at least as accurate, benefit from using MBFB over MBF-AVG.

Employing zero padding with PEAK, P-Pd, boosts the MBFB positioning accuracy by 1.7cm/3.2cm. While the zero pad operation does not increase the frequency resolution, the sinc-interpolation ‘combines’ (i.e., filters) the magnitudes of the closely-spread peaks originating from the variation in the instantaneous CF (over an FFT interval) to ensure a more accurate total spectral magnitude at the CF. Finally, P-PdF manages to shave an additional 1.5mm and 0.6mm of MBFB’s $p_{50}$ and $p_{90}$ with respect to P-Pd, whilst also improving the metrics of Tril and MBF-AVG. MBFB’s, MBF-AVG’s and Tril’s $p_{50}$/$p_{90}$ now equal, respectively, 5.0cm/10.6cm, 5.3cm/11.5cm and 7.9cm/15.6cm. P-PdF’s MBFB’s performance starts rivalling VLP’s (see Section 3.2.2 and Section 3.3.2).

Frequency-sliding correlation C-F betters SPECT, but not quite PEAK with a MBF-AVG $p_{50}$ performance of 8.3cm. C-F does come at a skyrocketing $p_{90}$. C-FPh improves upon C-F to also account for phase dependence. With steps of θ=30°, C-FPh diminishes the MBF-AVG $p_{50}$/$p_{90}$ to 7.3cm/16.0cm. However, for the practical parameters, C-FPh does not succeed in besting PEAK. This can (partly) be attributed to the coarseness of the phase and frequency stepping as can be seen at the top right of Figure 8d. Lastly, to round out this analysis, AR-P only effectuates limited improvements, in the range of a centimetre, over AR-S. AR-P’s limited performance can be attributed to the power spectral density estimation (slightly) altering the $P_{R,i}$-$d_{i}$ relation.

#### 3.3.2. Influence of Demodulation on VLP Rmse

Figure 10 also delineates ‘regular’ VLP’s FFT and correlation-based positioning performance. SPECT/PEAK FFT-based demodulation, i.e., configuration 10 dubbed *S*, shows that the MBFB, MBF-AVG and Tril algorithms in order achieve $p_{50}$/$p_{90}$ values of 3.5cm/6.8cm, 5.0cm/10.1cm and 5.3cm/9.6cm. As there is little to no impact of the zero padding, the corresponding results are omitted. In combination with sliding window correlation every θ=10° i.e., C-FPh with Δf=15Hz, MBFB exhibits the exact same $p_{50}$/$p_{90}$ values as for FFT-based VLP. The improved MBF-AVG accuracies now total 4.6cm/9.7cm. C-FPh does (slightly) augment the Tril inaccuracy.

Disregarding their equivalent MBFB performance, both demodulation techniques reach their minimal $p_{50}$ when applying MBF-AVG. The reported $p_{50}$/$p_{90}$ values hence allow ranking C-FPh slightly ahead of SPECT in terms of the attainable rMSE. For the sake of completeness, the C-F bar plot justifies the additional phase sliding of C-FPh. Finally, it was also verified that sine-based C-FPh and its square wave-based variant score comparable. Both exhibit the same MBFB $p_{50}$ and $p_{90}$. Square-wave C-FPh reduces the outliers (and $p_{90}$) of both MBF-AVG and Tril, compared to sine-based C-FPh (and SPECT). The reduction comes at the cost of a larger $p_{50}$ for MBF-AVG.

The principle conclusion of this entire Section 3.3 is that VLP, either with SPECT or C-FPh in conjunction with MBFB, effectuates a mere 1.5cm (29.2%) and 3.8cm (35.6%) improvement over uVLP’s best scores of 5.0cm/10.6cm (also obtained with MBFB). This highlights the localisation potential of uVLP, certainly when considering its economics.

#### 3.3.3. Sources of Localisation Error

**Figure 11:**
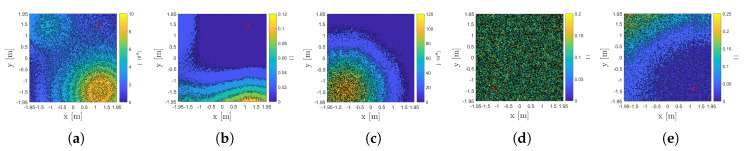
Per grid point normalised standard deviation $\sigma \left(I_{PD,i}\right)\;/\;\left(M\;\cdot \;P_{t,i}\;\cdot \;R_{P}\left(0\right)\right)$ of the AVG=10 segments’ $I_{PD,i}$ for various LEDs for (**a**) VLP and (**c**) uVLP PEAK. $\sigma \left(I_{PD,i}\right)\;/\;I_{PD,i}$ is also shown for (**b**) VLP, for uVLP (**d**) PEAK and (**e**) P-Pd. The red cross designates the LED’s location.

The main positioning degrading factors are identified to be LED interference (certainly for VLP, see Figure 11), the (limited) LED tilt present (i.e., due to the nonideal lab setup) [21], measurement errors, and noise. Once accurately measured, the tilt influence could be mitigated by incorporating it into the propagation model of the MBF-based techniques (Section 2.5.3). For VLP, LED interference does impede $I_{PD,i}$-based tilt estimation [35].

The first 4 degrading factors introduce (consistent) bias errors and impact the localisation accuracy. The latter, the noise influence, determines the localisation precision. Reverting to the set of AVG=10 per segment $I_{PD,i}$ values per grid point, allows to discern the portion of the positioning error that can be attributed to noise. Hereto, 2 metrics are computed per grid point with respect to (localisation with) the mean $I_{PD,i}$, namely the standard deviation on (1) $I_{PD,i}$ denoted by $\sigma \left(I_{PD,i}\right)$ and (2) the rMSE. The 50th and 90th percentile deviation on the localisation error during MBFB, with respect to the mean $I_{PD,i}$’s rMSE, is represented by $p_{50,err}$ and $p_{90,err}$.

##### Standard Deviation $\sigma \left(I_{PD,i}\right)$ on $I_{PD,i}$

Figure 11 depicts the spatial distribution of $\sigma \left(I_{PD,i}\right)$ normalised by $M\;\cdot \;P_{t,i}\;\cdot \;R_{P}\left(0\right)$ for (a) VLP and (c) uVLP PEAK/SPECT. It also plots the ratio $\sigma \left(I_{PD,i}\right)\;/\;I_{PD,i}$ for (b) VLP, for uVLP (d) PEAK and (e) P-Pd. Figure 11 shows both the location-dependence and the uVLP/VLP difference when it comes to $\sigma \left(I_{PD,i}\right)\;/\;\left(M\;\cdot \;P_{t,i}\;\cdot \;R_{P}\left(0\right)\right)$. Both FFT- and correlation-based VLP’s $\sigma \left(I_{PD,i}\right)$ is dominated by LED interference. Neighbouring (both in space and frequency) LEDs influence each other’s $I_{PD,i}$. For LED 4’s $I_{PD,4}$ ($f_{c,4}=4\;k\mathrm{Hz}$) shown in Figure 11a, LED 3 (with $f_{c,3}=2\;k\mathrm{Hz}$ and located at the bottom right) supplies the largest contribution.

During uVLP, no (dominant) interference contribution is present. Moreover, the (dominant) $\sigma \left(I_{PD,i}\right)\;/\;\left(M\;\cdot \;P_{t,i}\;\cdot \;R_{P}\left(0\right)\right)$ component varies with the employed demodulation technique as attested by Figure 11d,e. uVLP PEAK/SPECT’s $\sigma \left(I_{PD,i}\right)$ exhibits a strong proportional component to $I_{PD,i}$ (see Figure 11c,d). As the associated spatial sample variance $\sigma {\left(I_{PD,i}\right)}^{2}\;/\;I_{PD,i}^{2}$ profile follows a scaled Chi-squared distribution, Cochran’s theorem dictates that the underlying noise contribution can be modelled to be additive Gaussian with zero mean and with a variance amounting to the expectation of the sample variance. Hence, $\sigma \left(I_{PD,i}\right)\;/\;I_{PD,i}$ is location-independent. Nor $\sigma {\left(I_{PD,i}\right)}^{2}$ nor $\sigma {\left(I_{PD,i}\right)}^{2}\;/\;I_{PD,i}^{2}$ are chi-squared distributed for P-Pd (or for VLP). As can be expected from their associated localisation performance, VLP outranks in order uVLP P-Pd and PEAK/SPECT in terms of the absolute magnitude of the spatial expectation of $\sigma \left(I_{PD,i}\right)\;/\;\left(M\;\cdot \;P_{t,i}\;\cdot \;R_{P}\left(0\right)\right)$.

##### Localisation Precision

For uVLP PEAK/SPECT, $p_{50,err}$ and $p_{90,err}$ amount to 15.5cm and 21.6cm. uVLP P-Pd effectuates a significant $p_{50,err}$/$p_{90,err}$ reduction, the quantities now equalling 7.8cm/12.8cm. For FFT-based VLP, in order, the values are 1.3cm and 3.8cm. However, the positioning results of Section 3.3 are obtained on the averaged $I_{PD,i}$. Under assumptions (such as uncorrelatedness), the above numbers are reduced by a factor $\sqrt{AVG}$. Later, in Section 4, it is revealed that this is not necessarily the case.

It can be concluded that (1) VLP’s performance is mainly limited by inaccuracies (listed in the beginning of this section), that (2) a significant part of uVLP’s loss of accuracy (compared to VLP) is due to its significantly lower SNR and that (3) the superior (uVLP) demodulation techniques deliver a higher SNR.

##### Influence Segment Count for $I_{PD,i}$ Averaging

**Figure 12:**
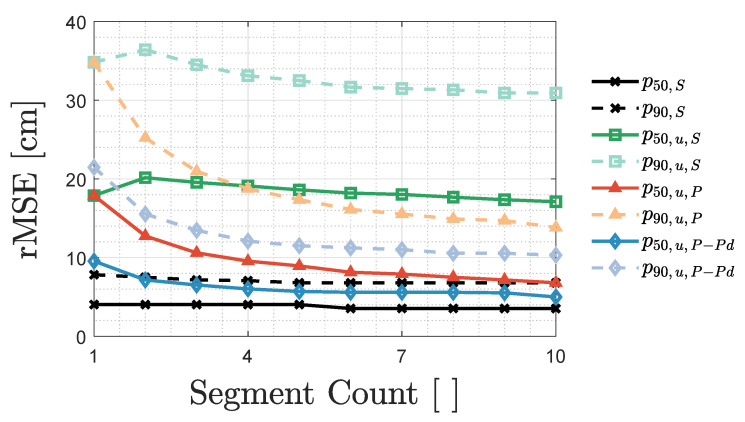
Influence on the $p_{50}$ and $p_{90}$ of MBFB-based VLP S, uVLP S, uVLP P and uVLP P-Pd of averaging the segments’ $I_{PD,i}$.

To further study the precision, Figure 12 depicts the $p_{50}$ and $p_{90}$ for VLP S, uVLP S, uVLP P and uVLP P-Pd as a function of how many segments’ $I_{PD,i}$ is averaged before positioning, i.e., the segment count. The segment count, ranging from 1 to 10, dictates how many of the AVG segments are employed for calibration and MBFB-based localisation. In this analysis, each segments’ length remains constant at $N_{a}=N_{s}\;/\;10$ with $N_{s}=256\;kS$ the total amount of samples recorded during the 1s measurement interval.

Figure 12 again highlights the significant improvement arising from utilising PEAK over SPECT in uVLP. The latter even, being plagued by the $f_{c,i}$ time variation mismatch, inadvertently increases the rMSE before reducing it, when increasing the segment count. The uVLP curves do not yet demonstrate convergence, meaning that uVLP’s performance can still be ameliorated by increasing the segment count coming at a latency cost.

For VLP S, MBFB’s discrete nature and small $\sigma \left(I_{PD,i}\right)$ allows achieving the same $p_{50}$ with 1 segment as with 5 segments. The accompanying $p_{90}$ of 5 segments meanwhile save 13.1% over the single segment’s $p_{90}$. At a segment count of 6, convergences starts to set in. Not measuring the other 4 segments, increases the update rate by 40%. VLP C-FPh coincides with S, except when a single segment is used (treated in the next Section 3.3.4).

#### 3.3.4. Influence of $N_{a}$ for uVLP

A location update rate of 1Hz is, depending on the application, insufficient, think, e.g., drone flight (combining a 1D Lidar height estimate with 2D (u)VLP) or the tracking of fast-moving vehicles such as forklift trucks. Hence, in this part, the positioning influence of a single segment’s length $N_{a}$ is investigated (i.e., no averaging is performed). The optimal $N_{a}$ will be lower bounded by the frequency separation $f_{S}\;/\;N_{a}$ needed to separate all $f_{c,i}$ values of the different LEDs.

**Figure 13:**
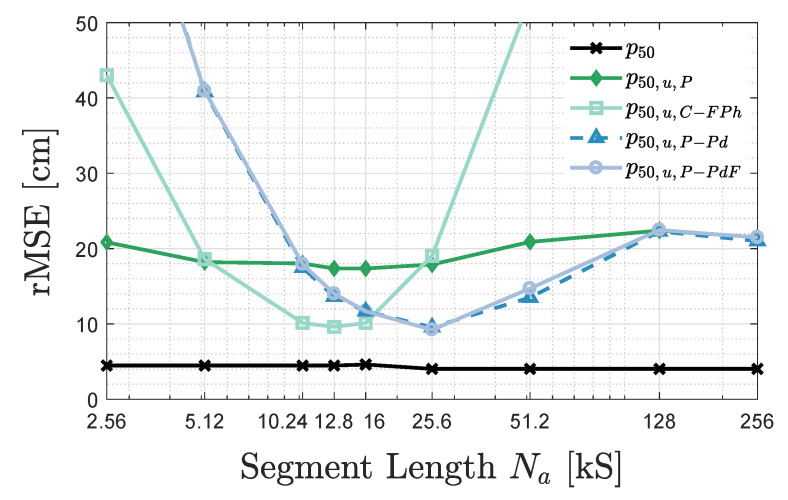
Influence of the sample size $N_{a}$ on the $p_{50}$ of MBFB for VLP (in black) and for uVLP PEAK, C-FPh, P-Pd and P-PdF.

Figure 13 shows the $p_{50}-N_{a}$ relation for VLP and uVLP PEAK, C-FPh, P-Pd and P-PdF. For VLP combining coherent sampling and stable $f_{c,i}$’s, $p_{50}$ gradually drops with increasing $N_{a}$ (until saturation) as a higher $N_{a}$ allows better resolving the $f_{c,i}$ mismatch (between the set and the actual $f_{c,i}$).

uVLP exhibits a different $p_{50}-N_{a}$ behaviour. Figure 13 portrays an unequivocal minimum for C-FPh, P-Pd and P-PdF that is located at 12.8kS (with $p_{50}=9.6\;cm$), 25.6kS (with $p_{50}=9.6\;cm$) and 25.6kS (with $p_{50}=9.2\;cm$), respectively. Comparing with Figure 10 allows to quantify the effect of not averaging $I_{PD,i}$, namely it augments the $p_{50}$ of P-Pd/P-PdF by approximately 85%. PEAK’s minimum is reached for both $N_{a}=12.8\;\mathrm{kS}$ and $N_{a}=16\;\mathrm{kS}$. Compared to Figure 10, PEAK’s $p_{50}$ substantially increases from 6.8cm to 17.9cm.

Interestingly, for uVLP, the optimal $p_{50}$ performance depends on the value of $N_{a}$ and is linked with a specific demodulation method. A too small $N_{a}$ hinders an accurate CF resolving, while a larger segment length holds (too) much temporal (magnitude) variation. This again shows the difficulty of dealing with the temporal variation in both frequency and magnitude of the CF.

This part is concluded by providing three examples elaborating on this difficulty. Intuitively, as C-FPh’s optimal $N_{a}$ amounts to 12.8kS, averaging all AVG=20 segments should better the $p_{50}$/$p_{90}$ of AVG=10 (with $N_{a}=25.6\;\mathrm{kS}$). However, $p_{50}$/$p_{90}$ growths of 1.2cm/0.7cm are incurred. A second example is found in uVLP PEAK with AVG=16 having both a lower $\sigma \left(I_{PD,i}\right)$ (over the AVG=16 segments) and a higher $p_{50}$/$p_{90}$ than AVG=10. Finally, taking the median instead of the mean $I_{PD,i}$, does not improve the positioning performance of uVLP and VLP. The accurate configurations, such as P-PdF and VLP, not necessarily exhibit a MBFB $p_{50}$/$p_{90}$ increase (PEAK does) when taking the median, but the $p_{50}$/$p_{90}$ belonging to Tril rises in all cases.

#### 3.3.5. Localisation Complexity

In localisation, it is not only the positioning performance parameter that matters. The complexity and thus latency is important as well. This part provides an approximation analysis. The total discretised $V_{PD,i}\left(t\right)$ sample length is $N_{s}=256\;kS$, resulting in a $N_{a}=N_{s}\;/\;AVG$ sample length per segment.

Assuming an FFT complexity of order $O\left(L\;\cdot \;\log_{2}\left(L\right)\right)$ (with *L* the FFT length) and a *N*-fold (for each LED) peak detect operation of order $O\left(Pk(L)\right)$, allows to discern the different demodulation algorithms based on complexity. $O\left(Pk(L)\right)$ depends on the $f_{c,i}$ stability and the frequency characteristics of (interfering) ambient LED sources. Pk(L) may be as simple as a binary search peak detection limited to 2Δf or more complex to also consider the peaks’ prominence (genre the ‘findpeaks’ function of MATLAB^®^).

In SPECT (or S), taking the mean of AVG FFT operations is succeeded by a single peak detect. The corresponding order of complexity amounts to $O\left(AVG\;\cdot \;N_{a}\;\log_{2}\left(N_{a}\right)\right)+$$O\left(Pk(N_{a})\right))$. In *S* and the subsequent, the $O\left(N_{a}\right)$ of the mean operation is assumed negligible (with respect to the dominant term). The order of zero padding (S-Pd) then augments to $O\left(AVG\;\cdot \;N_{a}\;\left(1+\log_{2}\left(N_{a}\right)\right)\right)+$$O\left(Pk(2\;\cdot \;N_{a})\right))$. For PEAK (or P), the order equals $O\left(AVG\;\cdot \;(N_{a}\;\log_{2}\left(N_{a}\right)+Pk\left(N_{a}\right))\right)$. In the previous, taking the mean $I_{PD,i}$ is neglected. P and P-Pd should be feasible for real-time applications (depending on the receiver’s constraints). The added complexity of the 3 step approach of P-PdF, however, will only be justified when the highest obtainable accuracies are required.

The complexity of the correlation-based demodulation routines also largely depends on the $f_{c,i}$ stability, and the frequency ($f_{step}$) and phase ($\theta_{step}$) granularity: O(C-F$)=N\;\cdot \;N_{a}\;\cdot \;2\;\Delta f\;/\;f_{step}$ and O(C-FPh$)=N\;\cdot \;N_{a}\;\cdot \;2\;\Delta f\;/\;f_{step}\;\cdot \;2\;\Delta \theta \;/\;\theta_{step}$. For the parameters considered in this manuscript O(C-FPh)≫O(P-Pd).

In conclusion, the optimal demodulation algorithm is governed by a trade-off regarding complexity and positioning accuracy. This trade-off is elaborated upon in Section 5, where various applications of (u)VLP are studied.

### 3.4. Influence of Positioning Algorithm

**Figure 14:**
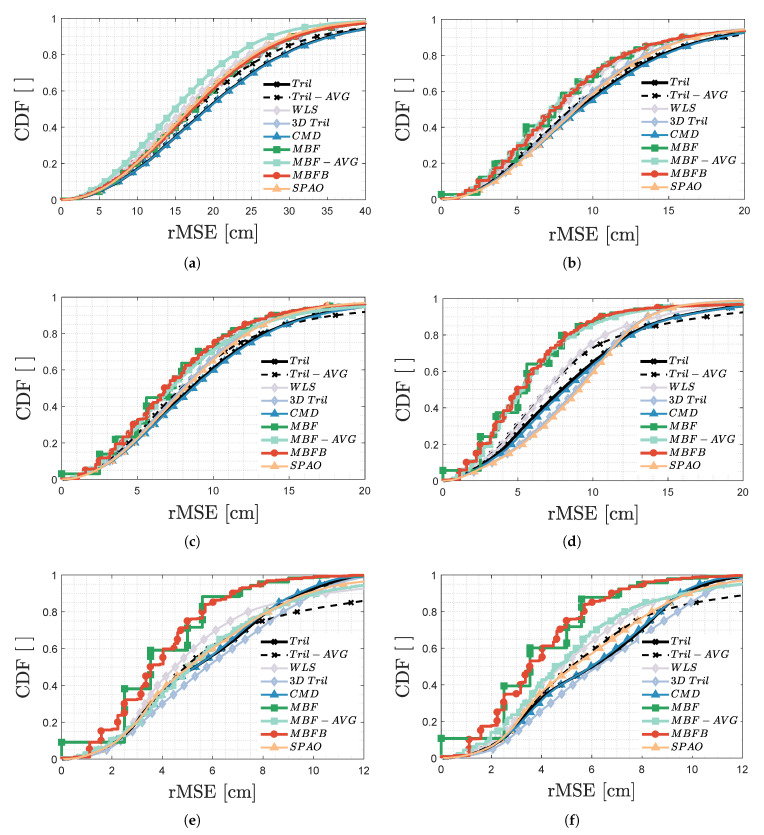
Cumulative distribution function (CDF) of the rMSE appertaining to the positioning algorithms of Section 2.5 in the case of uVLP in combination with (**a**) S, (**b**) C-FPh, (**c**) P and (**d**) P-PdF demodulation, and of VLP with (**e**) S and (**f**) C-FPh.

Figure 14 zooms in on the localisation aptitude of the different positioning algorithms of Section 2.5 related to both uVLP with (a) S, (b) C-FPh, (c) P and (d) P-PdF demodulation, and to VLP with (e) S and (f) C-FPh demodulation.

The cumulative distribution function (CDF) Figure 14a–d show that the positioning algorithms’ performance exhibits an SNR-dependence during uVLP. At low SNR and for uVLP SPECT, MBF-AVG with a $p_{50}=15.0\;cm$ significantly outperforms WLS with a $p_{50}=16.2\;cm$ (rank 2) and SPAO with a $p_{50}=16.9\;cm$ (rank 3). Tril and CMD display the worst $p_{50}$/$p_{90}$. When employing C-FPh or a PEAK-based demodulation, the MBF-family can be ranked as the best algorithms. It is a consequence of the MBF algorithms accounting for the receiver acceptance and the non-approximated LED radiation pattern [21]. The gap with the trilateration-family of algorithms widens furthermore with an increasing SNR. For P-PdF (and P), MBFB outscores, respectively, WLS and Tril by 2.0cm/3.8cm (0.3cm/0.7cm) and by 2.9cm/5cm (1.7cm/2.9cm) in terms of their $p_{50}/p_{90}$. As already stated in Section 3.3.1, MBFB takes the throne (over MBF and MBF-AVG) for P-PdF uVLP. In uVLP, Tril/CMD consistently display the loftiest $p_{50}$/$p_{90}$ rMSE values. WLS can be categorised as the best of the trilateration ‘enhancing’ algorithms due to its inherent robustness.

In the case of ‘regular’ VLP, MBFB and MBF still hold the top spots. In contrast to when uVLP is used, the algorithms considering the $I_{PD,i}$ of all *N* LEDs (namely MBF-AVG, Tril-AVG and WLS) show a distinctively inferior, and more rapidly stagnating CDF. This phenomenon can be attributed to the perceptual $I_{PD,i}$ found (see Figure 11b) being relatively more subject to LED interference than to other induced noise sources (e.g., the receiver chain’s input-referred current noise). MBF-AVG now manages to achieve a $p_{50}$ of 5.0cm/4.4cm and $p_{90}$ of 10.0cm/9.7cm in Figure 14e,f, only narrowly ducking below P-PdF uVLP’s 5.3cm and $p_{90}$ of 11.5cm rMSE. C-FPh’s lower relative $\sigma \left(I_{PD,i}\right)$ in the room’s corner, effectuates an MBF-AVG performance gain over FFT-based VLP. The gain does not manifest itself for MBFB. Its inherent robustness ensures that C-FPh and *S* exhibit the same $p_{50}$/$p_{90}$. Finally, during VLP, CMD and Tril rise to rank, respectively, 3th and 4 th in terms of the $p_{90}$.

Figure 14 thus illustrates that the optimal localisation algorithm depends on the utilised demodulation strategy. The MBF-based algorithms, and in particular MBFB with a tuned parameter *p*, are good choices (see Section 3.3.3). MBFB generalises better than MBF, when the measurement points do not overlap with the propagation model’s grid. While adequately performing at low SNR, 3D Tril and SPAO are not worth the additional effort if indeed no 3D location is required and (receiver) tilt is limited. CMD and Tril score comparable, with Tril being (slightly) better in conjunction with uVLP while CMD is with VLP.

## 4. Simulation Results

The previous Section 3 demonstrated the decimetre potential of uVLP-based indoor localisation for a (lab) VLP roll-out with a 2.25m perpendicular distance between the illumination and receiver plane. This simulation section extends the experimental results to also consider (1) the feasibility of uVLP when LEDs are suspended higher up (Section 4.1) and (2) the cost-saving effect of uVLP from not modifying the existing lighting infrastructure (Section 4.2).

To provide answers for these two viable research questions, the 4m by 4m VLP setup is virtualised. The simulation setup resembles Section 2.6, in i.a. the LED locations, and employs the propagation model of Section 2.5 with additive Gaussian noise characterised by a zero mean and ${\hat{\sigma }}^{2}$ variance. AVG=10 noise samples will be averaged. For generality, all LEDs are assumed to have both a uniform z-coordinate and a $M\;\cdot \;P_{t,i}\;\cdot \;R_{P}\left(0\right)$ (see Section 2.6) value.

### Noise Models

The simulations account for the spatial dependence of the noise contribution, i.e., of the sample standard deviation $\sigma \left(I_{PD,i}\right)$ reported in Section 3.3.3, by introducing three models that are denoted by (1) $\sigma_{u,\;P,\;M}$, (2) $\sigma_{u,\;Pd,\;M}$ and (3) $\sigma_{V,\;S,\;M}$.

(1) $\sigma_{u,\;P,\;M}$ represents uVLP PEAK/SPECT and models ${\hat{\sigma }}^{2}$ as the product of the expectation of all $MM\sigma {\left(I_{PD,i}\right)}^{2}\;/\;I_{PD,i}^{2}$ and $I_{PD,i}^{2}$. (2) $\sigma_{u,\;Pd,\;M}$ is the model derived for uVLP P-Pd that describes ${\hat{\sigma }}^{2}$ via $10^{\bar{\sigma }}\;\cdot \;{\left(M\;\cdot \;P_{t,i}\;\cdot \;R_{P}\left(0\right)\right)}^{2}$ with $\bar{\sigma }$ the result of evaluating a power law fit, $a\;\cdot \;d_{i}^{b}+c$ of $log_{10}\left(\sigma {\left(I_{PD,i}\right)}^{2}\;/\;{\left(M\;\cdot \;P_{t,i}\;\cdot \;R_{P}\left(0\right)\right)}^{2}\right)$ in function of the LED-PD distance $d_{i}$, at the grid point’s $d_{i}$. (3) $\sigma_{V,\;S,\;M}$ power law fits $log_{10}\left(\sigma {\left(I_{PD,i}\right)}^{2}\;/\;I_{PD,i}^{2}\right)$ to characterise VLP’s $\sigma \left(I_{PD,i}\right)$. It should be noted that $\sigma_{V,\;S,\;M}$ by virtue of modelling ${\hat{\sigma }}^{2}$ in terms of $d_{i}$ will still underestimate the LED interference contribution. The power law fits are obtained via the MATLAB^®^ curve fitting tool cftool.

These experimental noise models are compared to the standard case in which ${\hat{\sigma }}^{2}$ equals the expectation (i.e., spatial average) of $\sigma {\left(I_{PD,i}\right)}^{2}$: $\sigma_{u,\;P,\;G}$, $\sigma_{u,\;Pd,\;G}$ and $\sigma_{V,\;S,\;G}$. The models presume a dominant Gaussian input-referred rms current noise (i.e., corresponding to the receiver chain being the dominant noise contribution). However, it needs to be remarked that assuming the (dominant contribution to the) sample variance $\sigma {\left(I_{PD,i}\right)}^{2}$ following a (location-independent) scaled chi-squared distribution is not a valid hypothesis (see Section 3.3.3). The noise model names will also be used to describe the overarching simulation configuration.

### 4.1. Feasibility of (U)VLP in the Presence of Higher Ceilings

An important application domain for indoor localisation is found in the industrial or mobile warehouse environment [22], characterised by larger LED-receiver plane distances or heights. In this first study, the impact of the LED plane’s height on the (u)VLP rMSE is determined.

**Figure 15:**
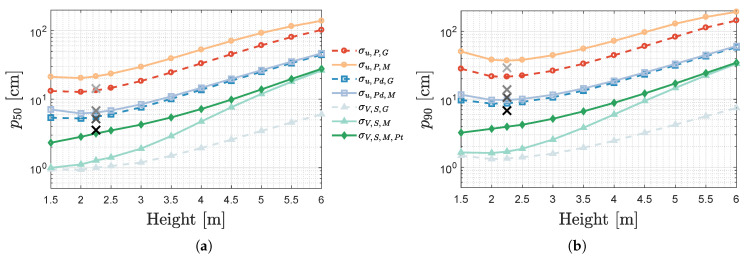
Influence of the perpendicular LED PD distance on (u)VLPs (**a**) $p_{50}$ and (**b**) $p_{90}$ rMSE for various noise models.

Figure 15 shows the (a) $p_{50}$ and (b) $p_{90}$ for the noise models previously introduced in conjunction with MBFB-based positioning. The curves of the other positioning algorithms display the same trends. Figure 15 demonstrates that the location-independent noise models ($\sigma_{u,\;P,\;G}$, $\sigma_{u,\;Pd,\;G}$ and $\sigma_{V,\;S,\;G}$) provide a lower bound for their location-dependent counterparts ($\sigma_{u,\;P,\;M}$, $\sigma_{u,\;Pd,\;M}$ and $\sigma_{V,\;S,\;M}$).

The $p_{50}$/$p_{90}$ values at a height equal to 2.25m allow comparing the simulation results with the experiments’ from Section 3. The black crosses in Figure 15 represent in order of blackness the $p_{50}$/$p_{90}$ of VLP, uVLP P-Pd, uVLP PEAK and uVLP SPECT. Concerning the two uVLP PEAK/SPECT configurations, $\sigma_{u,\;P,\;G}$ and $\sigma_{u,\;P,\;M}$, it can easily be remarked that they correspond well to uVLP SPECT rather than PEAK. As also visualised in Figure 12, PEAK’s rMSE and variance decrease more rapidly for AVG=10 than with AVG (compared to AVG=1). Hence, even $\sigma_{u,\;P,\;G}$, an underestimating $\sigma \left(I_{PD,i}\right)$-model, still overvalues PEAK’s $p_{50}$/$p_{90}$. uVLP’s P-Pd configurations better match the reality (and each other). The models both predict a less accurate $p_{50}$ and a more accurate $p_{90}$. $\sigma_{u,\;Pd,\;M}$’s $p_{90}$ totals to 9.8cm, a mere 8.6mm off the mark.

The $p_{50}$/$p_{90}-Height$ curves appertaining to uVLP are not monotonically increasing. uVLP’s $p_{90}$ reaches its minimal value at 2m, while for its $p_{50}$ it is found at 2.25m (except for $\sigma_{u,\;Pd,\;G}$ located at 2.25m). The $p_{50}$/$p_{90}$ at 1.5m is hindered by its higher irradiance angles inducing lower SNR’s further away from the LEDs.

For standalone uVLP, it is difficult to attain the $p_{90}\leq 10\;cm$ bound often required in navigation (of robotics) in industrial or warehouse-like environments, where ceiling heights are typically larger. In the $\sigma_{u,\;Pd,\;M}$ configuration, $p_{90}\leq 10\;cm$ is only reached within the range 1.9m to 2.4m. $p_{50}\leq 10\;cm$ is more easily obtainable. A height of about 3.3m can be covered. uVLP is able to achieve $p_{90}\leq 30\;cm$, another typical, but more lenient bound, up to 4.8m. The penultimate Section 5 of this manuscript translates these bounds into potential applications.

In VLP, applying either of the noise models leads to an underestimation of the real-life $p_{50}$/$p_{90}$. Not assuming $M\;\cdot \;P_{t,i}\;\cdot \;R_{P}\left(0\right)$ to be known, and effectuating the $P_{t,i}$ calibration (of Section 2.6) in the presence of $\sigma_{V,\;S,\;M}$ (i.e., LED interference), results in the curves designated by $\sigma_{V,\;S,\;M,\;Pt}$. $\sigma_{V,\;S,\;M,\;Pt}$ manages at least to bridge part of the gap to reality, by exhibiting a comparable (but still 4mm lower) $p_{50}$ whilst grossly underestimating the $p_{90}$ (compared to Figure 10). The disparity in $p_{90}$ originates from not sufficiently modelling the LED interference and other performance degrading factors (Section 3.3.3) present in the lab setup. For the other simulation configurations, calibration does not instil such a large accuracy decrease.

VLP’s $p_{90}\leq 10\;cm$ crash depth is found at a perpendicular LED-PD distance of 4.2m. However, VLP is able to provide $p_{90}\leq 30\;cm$ accurate positioning across the considered LED-PD range. Furthermore, the $p_{50}$/$p_{90}-Height$ relations of $\sigma_{V,\;S\;M}$ and $\sigma_{V,\;S\;M,\;Pt}$ are monotonically increasing with enlarging height.

### 4.2. (U)VLP and Cost-Savings

The introduction stated that a prime pull factor of (u)VLP is its cost-effectiveness arising from reusing the existing illumination infrastructure. VLP-enabling the lighting, however, not only requires a costly retrofit but also (approximately) halves the illuminance. The latter in turn requires the VLP roll-out to feature additional LED luminaries to account for the same maintained illuminance ${\bar{E}}_{m}$. In uVLP, the infrastructure is left ‘as is’.

**Figure 16:**
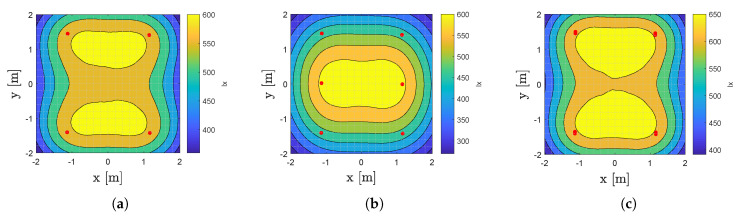
Illuminance distribution in the virtualised lab setup (**a**) during uVLP operation, (**b**) during VLP with 6 LEDs on 2 rails and (**c**) during VLP with 8 LEDs where an additional LED is placed 5cm north from each of the original LEDs.

This part illustrates uVLP’s cost-savings by visualising ${\bar{E}}_{m}$ found in the virtual lab setup during (uVLP) operation in Figure 16. Hereto, first the luminous flux $I_{max}$ of the LEDs’ during uVLP is computed, and rounded to 2 significant digits, to be 6000lm. $I_{max}$ is taken as the average of (1) the $I_{max}$ computed from VLP’s $P_{t,i}$[36] using the LEDs’ C0-C90 photometric diagram [21], Sharpe’s luminosity function [37] and the tabulated typical DC flux and (2) the $I_{max}$ that arise from translating the tabulated typical DC flux to the correct 1.2A LED current. Intuitively, $I_{max}$ during VLP would be half of $I_{max}$ exhibited in uVLP. However, due to the luminous flux - LED current relation being different during stable DC operation and pulse operation, VLP’s $I_{max}$ is larger (here 0.55 of uVLP’s $I_{max}$).

Figure 16a shows the spatial illuminance distribution during uVLP. ${\bar{E}}_{m}=549.4\;\mathrm{lx}$ and the uniformity $U_{0}$ equals 0.66. In VLP operation, ${\bar{E}}_{m}$ becomes 302.2lx and $U_{0}$ remains equal to 0.66. Figure 16b shows that suspending two additional (VLP) LEDs midway the lighting rails is not enough to restore ${\bar{E}}_{m}$ (see also the Zonal-Cavity Method [38]): ${\bar{E}}_{m}=505.3\;\mathrm{lx}$ and $U_{0}$ = 0.53. The 8 LED configuration of Figure 16c with ${\bar{E}}_{m}=604.3\;\mathrm{lx}$ and $U_{0}$ = 0.65 does satisfy the minimum ${\bar{E}}_{m}$ requirement.

## 5. (U)VLP and Potential Applications

Section 3 demonstrated uVLP’s capability in achieving a decimetre-like $p_{90}=10.6\;cm$ rMSE, albeit at a limited scale. Section 4 extended the experimental analysis to larger LED-PD distances, such as those found in a typical industrial or mobile warehouse hall. It also considered the roll-out’s illuminance, and highlighted the cost saving effect of not modulating the illumination LEDs (compared to VLP). The fact that uVLP does not necessitate a dedicated infrastructure, a lighting infrastructure that is furthermore omnipresent, also leads to a substantial cost-reduction with respect to the other indoor positioning systems on the market. Certainly, as only the to be tracked object needs to be equipped with a VLP-enabled receiver, which in itself is not of large cost. Table 1 lists some of the main conclusions and the benefits of rolling-out uVLP.

This Section 5 investigates the applicability of uVLP and VLP for several envisioned indoor localisation use cases. Indoor navigation of fast-moving automated guided vehicles (AGVs) or drones requires subdecimetre positioning estimates delivered with a low latency, effectively ruling uVLP systems out (see Figure 13). Compared to current (e.g., laser-based) navigation solutions, VLP may provide a cost-effective alternative when the LED-object suspension height does not exceed some 4m. uVLP, potentially supplemented with location tracking/filtering techniques [34], is not precluded from robot tracking however. Several tracking solutions employ laser-based positioning (with low refresh rates) to correct the inertial navigation (with encoders) of vehicles. Depending on the pre-existing LED deployment, uVLP is able to yield a $p_{90}\leq 10\;cm$ tracking on a limited scale, e.g., of a 1m tall AGV in an environment with a 3m ceiling height.

Other envisioned applications of uVLP entail, but are not limited to: (asset) tracking of, e.g., hospital beds, navigation with or to a car in a parking garage, and virtual and augmented reality. These applications generally only require a $p_{90}∼30\;cm$, which is certainly accomplishable for uVLP. However, uVLP systems will first need to evaluate a trade-off in latency (see Figure 12) and roll-out height (up to 4.8m). Luckily, with ample roll-out height and a minimal per-object roll-out cost, uVLP has a large market potential.

## 6. Conclusion and Future Work

This manuscript provided an in-depth study on the localisation performance, illuminance and applicability of (unmodulated) visible light positioning. It demonstrated that all tested constant current LED drivers exhibit a distinctive characteristic frequency (CF), which can serve as a LED demultiplexing feature in photodiode (PD)-based received signal strength (RSS) uVLP. Significantly reducing the cost, FFT-based uVLP only worsens the MBFB $p_{50}$ rMSE from VLP’s 3.5cm to 5.0cm, in the presence of 4 point source-like LEDs in the 4m × 4m room under consideration. Meanwhile, VLP ameliorates uVLP’s $p_{90}$ just from 10.6cm to 6.8cm. This allows to conclude that uVLP is able to ensure accurate localisation, albeit at the limited scale of 2.25m, without needing to retrofit the illumination infrastructure. While the measurements and ensuing simulation results did demonstrate uVLP’s limitations for low-latency industrial tracking applications, Section 5 discussed several exciting applications for uVLP-based localisation systems.

This paper did not cover all potential demodulation and filtering techniques. To boost uVLP’s positioning accuracy even more, it might be imperative to look at other methods. Accuracy improvements should also be searched in the location tracking, external sensor fusion (e.g., with wheel encoders) or machine learning domain. Other future work consists of studying uVLP’s positioning performance with non-point-source-like LEDs and comparing it with fluorescent uVLP. Subsequently, uVLP’s aptitude in industrial settings should also be experimentally investigated. There, dead reckoning and/or sensor fusion methods may be needed. The proneness of the CF to LED ageing is also work that remains.

## Figures and Tables

**Table 1:** Comparison of uVLP and ‘regular’ VLP.

Description	uVLP	VLP
Principle		
Frequency range	30–90 kHz	up to MHz
Modulation index	0%	50%
Average LED current	100%	50%
Luminous flux per LED	100%	55%
Accuracy		
Experimental $p_{50}$	5.0 cm	3.5 cm at 2.25 m
Projected $p_{50}$ in industry	>46.7 cm	>27.8 cm at 6 m
Cost		
Retrofitting effort	None	VLP-enabled LED driver
Transmitter-side cost	None	LED driver + new lamps for illuminance
Receiver-side cost	Equal	Equal

## Abbreviations

The following abbreviations and parameters are used in this manuscript:

**Acronym**	**Description**
*3D Tril*	3D extension algorithm of trilateration (*Tril*) [30]
*AGV*	Automated Guided Vehicle
*AR*	Autoregressive Yule-Walker PSD estimation-based (u)VLP
AVG	The number of segments $V_{PD}\left(t\right)$ is subdivided in, prior to demodulation, to average $V_{PD,i}$/$I_{PD,i}$
*BLE*	Bluetooth Low Energy
*CDF*	Cumulative Distribution Function
*CF*	Characteristic Frequency
*C-F*	Sliding-window correlation over frequency
*C-FPh*	Sliding correlation over frequency and phase
*CMD*	Cayley-Menger Determinant localisation [31]
*DALI*	Digital Addressable Lighting Interface
$d_{i}$	Distance between the receiver and LED *i*
${\bar{E}}_{m}$	Maintained Illuminance
$f_{0}$	Ground harmonic frequency of VLP (=500Hz)
$f_{c,i}$	LED *i*’s frequency: modulation frequency (VLP) or *CF* (uVLP)
*FDMA*	Frequency Division Multiplexing Access
*FFT*	Fast Fourier Transform
$f_{S}$	Sampling frequency (=256kHz)
$f_{step}$, 2Δf	Frequency step, range during sliding correlation
$I_{max}$	Luminous flux
$I_{PD,i}$	Photocurrent-based RSS value, obtained via demodulation and converted in $P_{R,i}$
$I_{PD,i\;}\left(t\right)$	LEDs’ individual photocurrent contribution
K,K≤N	(Sub)set of LEDs used in the positioning algorithm (selected in order of descending $P_{R,i}\;/\;P_{t,i}$)
*KNN*	(K-) Nearest Neighbours positioning
*LED*	Light-Emitting Diode
*LSOOP*	Light Signals of Opportunity
*MBF*	Model-Based Fingerprinting [22]
*MBF-AVG*	Positioning algorithm that takes the mean of the location estimates of *N* MBF runs on K=N−1 (see Section 2.5.3)
*MBFB*	Extension of *MBFB*, where the location estimate is taken to be the mean of all grid coordinates for which the cost function is smaller than the *p*th percentile of that cost function (*p* is a parameter)
$M\;\cdot \;P_{t,i}\;\cdot \;R_{P}\left(0\right)$	LED-PD gain: 0.04A (uVLP) and 5A (VLP)
*MUSIC*	MUltiple SIgnal Classification
*N*	Number of LEDs (=4)
$N_{a}$	Segment length of each of the AVG segments i.e., $N_{a}=N_{s}\;/\;AVG$
$N_{s}$	Total number of samples collected during 1s of $V_{PD}\left(t\right)$ (=256kS)
O()	Order of Complexity
O(Pk(L))	Complexity of the Peak Detect operation
$p_{50}/p_{75}/p_{90}$	Median/75th/90th percentile positioning rMSE
$p_{50,err}/p_{90,err}$	50th and 90th percentile of the standard deviation over AVG segments, on the rMSE during *MBFB*
*PD*	Photodiode
*-Pd*	Suffix denoting the zero padding procedure that doubles the photovoltage signal’s length
*-PdF*	Suffix denoting the zero padding procedure, which obtains a per LED FFT length that is a multiple of $f_{c,i}$ i.e., to try to ensure coherent sampling.
*PEAK* or *P-*	Name or Prefix of the demodulation method that AVG times averages a peak detected $V_{PD,i}$/$I_{PD,i}$ value per location update
$P_{R,i}$	Received radiant power of LED *i*
*PSD*	Power Spectral Density
$P_{t,i}$	Radiant flux of LED *i*
*PWM*	Pulse-Width Modulation
*RSS*	Received Signal Strength
*rMSE*	root-Mean-Square Error
${\hat{\sigma }}^{2}$	Variance of the additive Gaussian noise contribution, used in the simulation Section 4
$\sigma \left(I_{PD,i}\right)$	Spatial standard deviation on the measured $I_{PD,i}$
$\sigma_{u,\;P,\;G}$	uVLP *Peak* noise model where ${\hat{\sigma }}^{2}$ equals the expectation of $\sigma {\left(I_{PD,i}\right)}^{2}$
$\sigma_{u,\;P,\;M}$	uVLP *Peak* noise model dictating ${\hat{\sigma }}^{2}$ as the product of the expectation of all $\sigma {\left(I_{PD,i}\right)}^{2}\;/\;I_{PD,i}^{2}$ and $I_{PD,i}^{2}$
$\sigma_{u,\;Pd,\;G}$	uVLP *P-Pd* noise model where ${\hat{\sigma }}^{2}$ equals the expectation of $\sigma {\left(I_{PD,i}\right)}^{2}$
$\sigma_{u,\;Pd,\;M}$	uVLP *P-Pd* noise model dictating ${\hat{\sigma }}^{2}$ via $10^{\bar{\sigma }}\;\cdot \;{\left(M\;\cdot \;P_{t,i}\;\cdot \;R_{P}\left(0\right)\right)}^{2}$
$\sigma_{V,\;S,\;G}$	VLP *SPECT* noise model where ${\hat{\sigma }}^{2}$ equals the expectation of $\sigma {\left(I_{PD,i}\right)}^{2}$

$\sigma_{V,\;S,\;M}$	VLP *SPECT* noise model characterised by a power law fit of $log_{10}\left(MM\sigma {\left(I_{PD,i}\right)}^{2}\;/\;I_{PD,i}^{2}\right)$
$\sigma_{V,\;S,\;M,\;Pt}$	$\sigma_{V,\;S,\;M}$ with $P_{t,i}$ calibration
*SNR*	Signal-to-Noise-Ratio
*SPAO*	Simultaneous Positioning and Orientating [32]
*SPECT* or *S-*	Name or Prefix of the demodulation method that AVG times averages the FFT-spectrum before peak detecting $V_{PD,i}$/$I_{PD,i}$ per location update
SQ	Angular acceptance model of [21]
θ	Phase angle during sliding window correlation
$\theta_{step}$/2Δθ	Phase Step/Range during sliding window correlation
*Tril*	basic Trilateration Algorithm [28]
*Tril-AVG*	Positioning algorithm that takes the mean of the location estimates of *N* Tril operations on K=N−1 (see Section 2.5.1)
$U_{0}$	Illuminance Uniformity
*uVLP*	Unmodulated Visible Light Positioning
*UWB*	Ultra-wideband
*VLP*	Visible Light Positioning
$V_{PD,i}$	Photovoltage-based RSS value, obtained via demodulation and converted in $P_{R,i}$
$V_{PD,i}\left(t\right)$	Individual photovoltage contribution of LED *i*
$V_{PD}\left(t\right)$	Total photovoltage time domain signal
*WLS*	Weighted Linear Squares trilateration based on singular value decomposition [29]
$\left(x_{S,i},\;y_{S,i},\;z_{S,i}\right)$	LED *i*’s coordinates
$\left(x_{u},y_{u},z_{u}\right)$	Unknown receiver position
